# The Cardiorespiratory Network in Healthy First-Degree Relatives of Schizophrenic Patients

**DOI:** 10.3389/fnins.2020.00617

**Published:** 2020-06-16

**Authors:** Steffen Schulz, Jens Haueisen, Karl-Jürgen Bär, Andreas Voss

**Affiliations:** ^1^Institute of Innovative Health Technologies (IGHT), University of Applied Sciences, Jena, Germany; ^2^Institute of Biomedical Engineering and Informatics, Ilmenau University of Technology, Ilmenau, Germany; ^3^Department of Psychosomatic Medicine and Psychotherapy, Jena University Hospital, Jena, Germany

**Keywords:** cardiorespiratory coupling, Network Physiology, partial directed coherence, transfer entropy, schizophrenia, relatives

## Abstract

Impaired heart rate- and respiratory regulatory processes as a sign of an autonomic dysfunction seems to be obviously present in patients suffering from schizophrenia. Since the linear and non-linear couplings within the cardiorespiratory system with respiration as an important homeostatic control mechanism are only partially investigated so far for those subjects, we aimed to characterize instantaneous cardiorespiratory couplings by quantifying the casual interaction between heart rate (HR) and respiration (RESP). Therefore, we investigated causal linear and non-linear cardiorespiratory couplings of 23 patients suffering from schizophrenia (SZO), 20 healthy first-degree relatives (REL) and 23 healthy subjects, who were age-gender matched (CON). From all participants’ heart rate (HR) and respirations (respiratory frequency, RESP) were investigated for 30 min under resting conditions. The results revealed highly significant increased HR, reduced HR variability, increased respiration rates and impaired cardiorespiratory couplings in SZO in comparison to CON. SZO were revealed bidirectional couplings, with respiration as the driver (RESP → HR), and with weaker linear and non-linear coupling strengths when RESP influencing HR (RESP → HR) and with stronger linear and non-linear coupling strengths when HR influencing RESP (HR → RESP). For REL we found only significant increased HR and only slightly reduced cardiorespiratory couplings compared to CON. These findings clearly pointing to an underlying disease-inherent genetic component of the cardiac system for SZO and REL, and those respiratory alterations are only clearly present in SZO seem to be connected to their mental emotional states.

## Introduction

Schizophrenia represents a mental disorder along with increased cardiovascular mortality rate, shorter life expectancy, higher risk of developing cardiovascular disease (CVD) in proportion to the general population (Hennekens et al., 2005; McGrath et al., 2008; Laursen et al., 2014). One reason in schizophrenia, besides others (Straus et al., 2004; Hennekens et al., 2005; Ringen et al., 2014), seems to be an unbalanced autonomic nervous system (ANS) during the acute psychosis state quantified by analyses heart rate variability (HRV) and respiratory variability (RESPV). Different studies suggested as a major contributing factor the unbalanced sympathovagal balance for schizophrenic patients, as well as for their healthy first-degree relatives (Valkonen-Korhonen et al., 2003; Bär et al., 2005, 2007; Chang et al., 2009; Schulz et al., 2013c). However, investigations of respiration and cardiorespiratory couplings is becoming more of interest in medicine and research (Peupelmann et al., 2009; Bär et al., 2012; Schulz et al., 2012a,b, 2013b, 2014, 2018) for schizophrenia since respiration plays a major part homeostatic regulatory control processes. As far as we know there exist only a few investigations dealing with causal couplings quantifying the coupling strengths and coupling directions in these patients. The field of Network Physiology aiming to identify and quantify the dynamics within the (patho)physiological network with their different sub-networks and their interactions between them (Bashan et al., 2012) but seems to be a promising multivariate concept to describe the cardiorespiratory system. Moreover, Network Physiology quantifies healthy and diseased states investigating the coupling between systems and sub-systems by determining structural, dynamical and regulatory changes. These new concepts allow getting a better understanding of the complexity of physiological as well as pathophysiological processes in health and disease by linking genetic and subcellular levels with intercellular coupling pathways between integrated systems and subsystems (Ivanov et al., 2016).

Studies investigating HRV generally showed an altered sympathovagal balance pointing to dysregulation of heart rate for schizophrenic patients and partially their first-degree healthy relatives (Toichi et al., 1999; Valkonen-Korhonen et al., 2003; Bär et al., 2005, 2007, 2010; Castro et al., 2009; Chang et al., 2009; Voss et al., 2010). The pattern of an unbalanced ANS (heart rate) in schizophrenic patients and their relatives seem to hallmark a disease-inherent genetic feature of this disease. Busjahn et al. (1998) highlighted that there exist a genetic dependency of HRV indices. Studies analyzing respiration and cardiorespiratory couplings in schizophrenia are exclusive (Peupelmann et al., 2009; Bär et al., 2012; Schulz et al., 2012a,b, 2013b, 2015b, 2018) and demonstrated significantly altered dynamic and variability of respiration as well as impaired cardiorespiratory couplings for schizophrenic patients but not for their healthy first-degree relatives. Respiration is regulated in the brain stem primarily for metabolic and homeostatic purposes, it also constantly reacts to changes in emotions (Homma and Masaoka, 2008). It seems that the altered psychotic states of schizophrenic patients compared to healthy subjects have a great influence on their cardiorespiratory system characterized by an interplay of different linear and non-linear subsystems (Voss et al., 2009). The respiratory sinus arrhythmia (RSA) occupies an important part of cardiorespiratory couplings. RSA describes the rhythmic fluctuation of heart rate in proportion to respiration. Under normal physiological conditions RSA characterizes changes between inspiratory heart rate acceleration and expiratory heart rate deceleration (Eckberg, 2003). Studies have actually shown that the coupling between cardiovascular system and respiration is strongly non-linear (Novak et al., 1993). For the analysis of the cardiorespiratory system as a complex physiological regulatory network, a variety of methods have been proposed (Schulz et al., 2013a; Bartsch et al., 2015; Faes et al., 2015; Liu et al., 2015; Ivanov et al., 2016) basing on Granger causality, phase synchronization, entropies, non-linear prediction, symbolization, and time delay stability (TDS) (Schulz et al., 2018).

Investigating the coupling between heart rate and respiration could provide potential clinically insights into (patho)physiological autonomic processes in schizophrenia and their relatives. In contrast to our preliminary work in this field, we have applied a pool of different coupling methods from the time and frequency domain that can quantify both linear and non-linear causal couplings. This will allow us to gain more insight into the regulatory processes of the cardiorespiratory system, which will provide a better understanding of how individual systems interact with each other in a healthy and explored state. This study aimed to quantify instantaneous cardiorespiratory couplings in schizophrenic patients and their healthy first-degree relatives. Therefore, multivariate linear and non-linear causal coupling approaches [normalized short time partial directed coherence, multivariate transfer entropy, cross conditional entropy, and respiratory sinus arrhythmia (peak-to-valley)] were applied determining causal coupling strengths and directions. We speculate that these new findings are important for a full understanding of (patho)physiological regulatory processes and possibly may help to improve treatment strategies in schizophrenia and identify those patients at increased risk for cardiovascular disease accompanied by ANS dysfunction.

## Materials and Methods

### Subjects

Twenty-three untreated patients suffering from paranoid schizophrenia (SZO), 20 healthy first-degree relatives (REL) and 23 healthy controls subjects (CON) (age–gender matched) (Table 1) were enrolled in this pilot study. Patients were included only when they had not taken any medication for at least 8 weeks. From all participants the serum drug levels were checked for legal drugs (e.g., antipsychotics, antidepressants, and benzodiazepines) and illegal drugs (e.g., cannabis). In accordance with the inclusion criteria, only subjects with negative results were included in the study. Paranoid schizophrenia was diagnosed when patients fulfilled DSM-IV criteria [Diagnostic and statistical manual of mental disorders, 4th edition. Psychotic symptoms (positive and negative) were quantified using the Positive and Negative Syndrome Scale (PANSS (Kay et al., 1987)]. The semi-structured clinical interview SCID-1 was used for patients to approve the clinical diagnosis. Control subjects were recruited from hospital staff, medical students and the local community. From all healthy control subjects and relatives interview and clinical investigation were performed to rule out any psychiatric or other disease or disruptive medication. Additionally for all controls the Structured Clinical Interview SCID II and a personality inventory (Freiburger Persönlichkeitsinventar) were applied to detect personality traits or disorders that could affect autonomic function (LeBlanc et al., 2004), and if present they were not included in this study.

**TABLE 1:** Clinical and demographic data of the study population.

**Data**	**Healthy controls subjects (CON)**	**Healthy first-degree relatives (REL)**	**Schizophrenic patients (SZO)**
Number of participants	23	20	23
Gender (male/female)	13/10	12/8	12/11
Age (mean ± std in years)	30.3 ± 9.5	31.7 ± 10.7	30.4 ± 10.3
PANSS, mean (min-max)	n.a.	n.a.	85.7 (43–124)
SANS, mean (min-max)	n.a.	n.a.	49.6 (14–81)
SAPS, mean (min-max)	n.a.	n.a.	60.9 (6–108)

**Psychotic symptoms for acute schizophrenia were quantified using the Scale for the Assessment of Positive Symptoms (SAPS) and negative symptoms (SANS) and positive and negative syndrome scales (PANSS); n.a., not applicable*.*

The written informed consent to a protocol approved by the local ethics committee of the Jena University Hospital (ethics committee number: 1190-09/03) was provided by all participants. This study complies with the Declaration of Helsinki.

### Data Recordings and Pre-processing

A short-term ECG (1,000 Hz) and synchronized calibrated respiratory inductive plethysmography signal (Bär et al., 2012) (LifeShirt^®^, VivoMetrics, Inc., Ventura, CA, United States) were recorded for 30 min under resting conditions [between 3 and 6 p.m. in a quiet room which was kept comfortably warm (22–24°C)] after 10 min rest in supine position. Subjects were asked not to talk, to relax and to breathe normally during the recording. For the further analyses from the raw data.

– Time series of successive beat-to-beat intervals (BBI, msec) and
– Time series of respiratory frequency (RESP, sec) as the time intervals between consecutive breathing cycles were automatically extracted.

These time series were afterward adaptively filtered (Wessel et al., 2000) to exclude and interpolate ventricular premature events and/or artifacts to obtain normal-to-normal beat time series (NN). Linear interpolation procedure was applied to filtered time series (BBI, RESP) for synchronization and resampling (2 Hz).

### Basic Data From the Heart Rate and Respiration

Basic indices from heart rate and respiration were determined as:

– meanNN: mean value of the NN intervals of BBI (msec), and RESP (sec) as respiratory cycle length;
– sdNN: standard deviation of the NN intervals of BBI (msec), and RESP (sec);
– HR: basic heart rate as the number of heart beats per minute (1/min), and
– BF: breathing frequency as the number of breaths per minute (1/min).

### Coupling Analyses

Different approaches can be used for the quantification of linear and non-linear cardiorespiratory couplings (Schulz et al., 2013a). In this study, we analyzed the coupling between BBI and RESP applying the linear normalized short-time partial directed coherence (NSTPDC) (Adochiei et al., 2013), the linear/non-linear multivariate Transfer Entropy (MuTE) (Montalto et al., 2014) and the non-linear cross conditional entropy (CCE) (Porta et al., 1999) approaches as well as the respiratory sinus arrhythmia (RSA).

#### Normalized Short-Time Partial Directed Coherence

NSTPDC represents an enhancement of the traditional partial directed coherence (PDC) (Baccala and Sameshima, 2001) approach assessing linear Granger causality in the frequency domain quantifying direct and indirect couplings within a set of multivariate time series. The fundamental basis of the NSTPDC is the time-variant partial directed coherence approach [tvPDC, π*_*xy*_*(*f*,*n*)] is allowing to determine causal short-term couplings between non-stationary time series at certain frequency *f* applying a window function (*n* is the number of windows) (Milde et al., 2011). An *m*-dimensional autoregressive (AR) model is used to calculate NSTPDC indices. The optimal model order *p*_opt_ was determined by the stepwise least squares algorithm (Neumaier and Schneider, 2001) and the Schwarz’s Bayesian Criterion (SBC) (Schneider and Neumaier, 2001). The coupling direction between two time series, *x* and *y*, (e.g., BBI and RESP) was determined by a coupling factor (CF) which is determined by the quotient of π*_*xy*_*(*f*,*n*) and π*_*yx*_*(*f*,*n*).

(1) $$\text{CF}=\frac{\frac{1}{n}\,\sum \pi_{x\,y}\,\left(f,n\right)}{\frac{1}{n}\,\sum \pi_{y\,x}\,\left(f,n\right)}$$

$$\bar{a}=\frac{1}{n}\,\sum \pi_{x\,y}\,\left(f,n\right)$$

$$\bar{b}=\frac{1}{n}\,\sum \pi_{y\,x}\,\left(f,n\right)$$

The results of CF were normalized and result in the normalized factor (NF), which characterizes the coupling direction.

$$\text{max}\,\left(\bar{a},\bar{b}\right)$$

$$\text{NF}=\left\{\begin{array}{c} 2,if\left(max=\bar{a}\&\frac{\bar{a}}{\bar{b}}>5\right)\;\\ 1,if\left(max=\bar{a}\&2<\frac{\bar{a}}{\bar{b}}\leq 5\right)\\ 0,if\left(max=\bar{a}\&0\leq \frac{\bar{a}}{\bar{b}}\leq 2\right) \end{array}\right\}\,\text{and}$$

(2) $$\text{NF}=\left\{\begin{array}{c} -2,if\left(max=\bar{b}\&\frac{\bar{b}}{\bar{a}}>5\right)\;\\ -1,if\left(max=\bar{b}\&2<\frac{\bar{b}}{\bar{a}}\leq 5\right)\\ 0,if\left(max=\bar{b}\&0\leq \frac{\bar{b}}{\bar{a}}\leq 2\right) \end{array}\right\}$$

Thereby, NF (NF = {−2, −1, 0, 1, 2}) determinates the causal coupling direction between the two time series (*x*_BBI_ and *y*_RESP_) as a function of frequency *f*.

Coupling direction:

– NF = {−2| 2} (where −2 denotes *y*_RESP_ as driver, +2 denotes *x*_BBI_ as driver): Strong unidirectional coupling;
– NF = {−1.5– < −2} or NF = {1.5–<2}: Weak unidirectional coupling;
– NF = {−1| 1} (−1 denotes *y*_RESP_ as driver, +1 denotes *x*_BBI_ as driver): Strong bidirectional coupling;
– NF = {−0.5– < −1} or NF = {0.5–<1}: Weak bidirectional coupling, and
– NF = 0: Equal influence in both directions and/or no coupling with respect to coupling strengths (If both area indices have equal values greater than zero, there is equal influence in both directions; if both area indices have equal values but are zero, there is no coupling).

Coupling strength:

In each window (*f* = 0–2 Hz) an area is made up of CF allowing to assess the coupling strength. For *x*_BBI_ and *y*_RESP_ these areas are:*A*_*BBI→RESP*_ and *A*_*RESP→BBI*_ [a.u.]. The values of these area indices ranges between 0 and 1 [0,1]. Thereby, 1 points to that from *x* all information is transferred (→) toward *y* (*A*_*x→y*_ = 1). Hamming window with a length of 120 samples and a shift of 30 samples per each iteration step was applied. To ensure scale-invariance all time series were normalized to zero mean and unit variance (Schulz et al., 2015a).

#### Multivariate Transfer Entropy

Transfer Entropy (TE) introduced by Schreiber (Schreiber, 2000) is able to quantify linear as well as non-linear information transfer between time series, to detect driver-response-relationships, and to assess asymmetries between information transfers. TE has the big advantage that it is “model-free” approach (Schulz et al., 2013a) making TE very sensitive to any types of dynamical information transfer. Montalto et al. (2014) introduced the Multivariate Transfer Entropy (MuTE) as a MATLAB toolbox with different entropy estimators to transfer the classical TE from a bivariate approach into a multivariate approach. The coupling strength of a multivariate set of time series can be determined as:

(3) MuTEXY→

with information transfer from *X* toward (→) *Y*, or vice versa. In this study we wanted to quantify non-linear couplings within the cardiorespiratory system with high specificity and sensitivity, therefore, we applied the non-uniform embedding (NN NUE) technique with the nearest neighbor estimator shown to be most suitable to detect non-linearities with high specificity and sensitivity (Montalto et al., 2014).

#### Cross Conditional Entropy

Porta et al. (1999) introduced the cross conditional entropy (CE*_*x/y*_*) based on the conditional entropy (CE). Thereby, CE*_*x/y*_* determines the level of coupling between the two time series *x* and *y*,

(4) $${\text{CE}}_{x|y}=-\sum_{L-1}p\,\left(y_{L-1}\right)\,\sum_{t|L-1}p\,\left(\frac{x\,\left(t\right)}{y_{L-1}}\right)\,\log p\,\left(x\,\left(t\right)/y_{L-1}\right)$$

with the pattern length *L*, the joint probability *p*(*y*_*L*__–__1_) of the pattern *y*_*L*__–__1_(*t*) and the conditional probability *p*(*x*(*t*)/*y*_*L*__–__1_) of the sample *x*(*t*), given that the pattern *y*_*L*__–__1_. CE*_*x/y*_* assesses the amount of information contained in the sample *x*(*t*) in the case that the pattern of *L*−1 samples of *y*_*L*__–__1_(*t*) is existing. Moreover, CE*_*x/y*_* quantifies causality by determining direct couplings regarding to cross-prediction approaches.

Finally, an uncoupling function $\bar{\text{UF}}$ can be estimated that determines the information content that is transferred between two time series (Porta et al., 1999). Here, we calculated the ${\bar{\text{UF}}}_{x,y}$ between HR and BF as ${\bar{\text{UF}}}_{\mathrm{HR},\mathrm{BF}}$. The larger $\bar{\text{UF}}$, the more decoupled the two time series are ($\bar{\text{UF}}=1$, HR und BF are completely independent from each other).

#### Respiratory Sinus Arrhythmia

Respiratory Sinus Arrhythmia (RSA) is used as an index of cardiac parasympathetic activity derived by heart rate changes (BBI) which correspond to inspiration and expiration (Grossman et al., 1990a). RSA is characterized by the shortening of heart rate intervals (BBI) during inspiration and the lengthening of heart rate intervals during expiration. In this study, we assess RSA in the time domain applying the peak-to-valley approach (RSA_P__2__V_, msec). The LifeShirt^®^ automatically estimated RSA using the peak-to-valley approach for each breathing cycle (Grossman et al., 1990b).

### Surrogate Data

To evaluate the significance of the cardiorespiratory couplings between CON and SZO as well as REL a surrogate data approach was applied (Schreiber and Schmitz, 2000). Here, from all original time series 20 independent surrogates were derived for each schizophrenic patient (SZOs), each relative (RELs), and each healthy control (CONs). The temporal structure within the original time series was destroyed by randomly permuting each sample to derived new surrogate time series. Afterward, we tested if significant couplings between the original time series were confirmed by the surrogate data. Therefore, a statistical defined coupling threshold level *t*_s_ (defined as the mean + 2^∗^SD of the resultant distribution derived from all surrogates SZOs, RELs, and CONs) was introduced. Significant valid couplings were present if no significant differences between two surrogate groups exist and if couplings (original data) were higher than *t*_su_.

In addition, a second surrogate approach was applied by generating random phase surrogates to test for non-linearity in the data. This surrogate approach is known as phase randomization and preserves linear behavior (i.e., the power spectrum/autocorrelation) but destroys any non-linear behavior. Preserving the power spectrum while randomizing the Fourier phases of the data providing surrogates in which any non-linear structure is destroyed (Lancaster et al., 2018).

### Statistics

For the statistical evaluation of the results between SZO, REL and CON first the Kruskal–Wallis test followed by the *post hoc* non-parametric exact two-tailed Mann–Whitney *U*-test in combination with the Kolmogorov–Smirnov test (check for equal distributions) (SPPS 21.0) were applied. The significance level was set to *p* < 0.01, and for highly significant different to *p* < 0.004 (Bonferroni–Holm adjustment). In order to check, if effects size have a relevant influence, effect sizes based on Cohen’s *d* were applied to describe the magnitude of the differences between the groups. The most popular effect size measure is Cohen’s *d* (Cohen, 1988).

Results were expressed in median and 25 and 75% percentiles.

## Results

The Kruskal–Wallis test revealed for all indices, with the exception of sdNN__RESP_, significant differences (*p* < 0.01) between all three groups.

### Patients Suffering From Schizophrenia vs. Healthy Subjects

Basic data from HR analysis revealed highly significant differences between SZO and CON. SZO showed shortened mean value of the NN intervals (meanNN__BBI_) and reduced variability (sdNN__BBI_) and higher HR (Table 2).

**TABLE 2:** Results of heart rate- and respiratory variability and cardiorespiratory coupling analyses to differentiate between patients suffering from paranoid schizophrenia (SZO), healthy first-degree relatives (REL), and healthy control subjects (CON).

	**Index**	**CON vs. SZO**	**Cohen’s *d***	**CON vs. REL**	**Cohen’s *d***	**SZO vs. REL**	**Cohen’s *d***	**CON**			**REL**			**SZO**		
								**Median**	**[25–75]**		**Median**	**[25–75]**		**Median**	**[25–75]**	
Cardiac	HR	***	†⁣†⁣†	***	†⁣†⁣†	n.s	†⁣†⁣†	62.4	56.7	70.8	72.8	69.8	80.0	80.9	71.5	88.6
	meanNN__BBI_	***	†⁣†⁣†	***	†⁣†⁣†	n.s	†⁣†⁣†	962.1	847.9	1058.2	824.2	750.2	859.2	741.4	677.4	839.1
	sdNN__*BBI*_	***	†⁣†⁣†	n.s	†⁣†⁣†	n.s	†⁣†	66.2	49.4	75.3	47.5	35.1	55.1	43.7	27.9	57.4
Respiratoy	BF	***	†⁣†⁣†	n.s	†⁣†⁣†	n.s	†⁣†⁣†	14.9	13.2	16.8	16.0	14.2	18.7	20.3	15.5	23.2
	meanNN__*RESP*_	***	†⁣†⁣†	n.s	†⁣†	n.s	†⁣†⁣†	4.0	3.6	4.5	3.8	3.2	4.2	2.9	2.6	3.9
	sdNN__*RESP*_	n.s	–	n.s	–	n.s	–	0.78	0.56	1.23	0.49	0.42	0.87	0.70	0.38	1.27
Couplings	NF	***	†⁣†⁣†	n.s	†⁣†⁣†	***	†⁣†⁣†	–1.9	–2.0	–1.9	–1.8	–1.9	–1.6	–1.3	–1.7	–0.8
	*A*_BBI → RESP_	***	†⁣†⁣†	n.s	†⁣†⁣†	n.s	†⁣†⁣†	0.04	0.03	0.06	0.06	0.04	0.08	0.07	0.06	0.10
	*A*_RESP → BBI_	***	†⁣†⁣†	n.s	†⁣†	***	†⁣†⁣†	0.46	0.35	0.53	0.39	0.33	0.46	0.27	0.19	0.35
	MuTE_BBI → RESP_	**	†⁣†⁣†	n.s	†⁣†	n.s	†⁣†	0.000	0.000	0.011	0.005	0.000	0.015	0.012	0.000	0.018
	MuTE_RESP → BBI_	***	†⁣†⁣†	n.s	†⁣†	n.s	†⁣†⁣†	0.047	0.033	0.061	0.043	0.011	0.049	0.011	0.000	0.026
	Ū F̄_*x,y*_	***	†⁣†⁣†	n.s	†	***	†⁣†⁣†	0.10	0.08	0.13	0.09	0.07	0.14	0.14	0.11	0.19
	RSA_P__2__V_	***	†⁣†⁣†	**	†⁣†⁣†	**	†⁣†⁣†	123.0	58.8	201.9	58.9	31.6	98.3	30.8	14.8	51.4

BBI, beat-to-beat intervals; RESP, time intervals between consecutive breathing cycles; HR, heart rate; BF, breathing frequency; A, area from NSTPDC for identifying the coupling strength; MuTE, multivariate Transfer Entropy; ${\bar{\text{UF}}}_{H\,R,B\,F}$, RSA, respiratory sinus arrhythmia; _*P*__2__*V*_, peak-to-valley; p, univariate significance level: **<0.01, ***<0.004; n.s., not significant; Cohen’s d: ^†^0.1—0.3: small effect, ^†⁣†^0.3—0.5: medium effect, ^†⁣†⁣†^0.5 and higher: strong effect).

Variability analyses of RESP showed a reduced (significant) mean respiratory cycle length (meanNN__*RESP*_) and consequently an increased breathing frequency (BF) in SZO compared to CON (Table 2).

Cardiorespiratory analysis revealed significant differences between the couplings in SZO than CON (Table 2).

**FIGURE 1:**
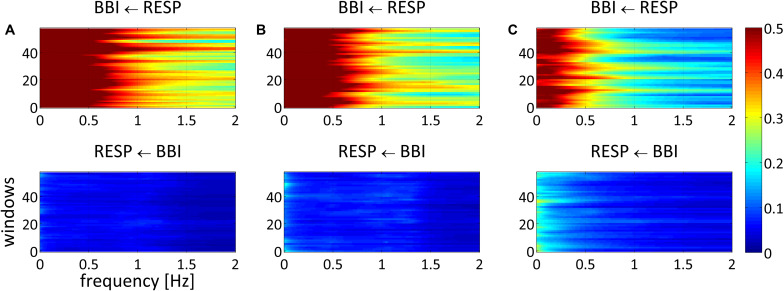
Averaged NSTPDC plots for cardiorespiratory coupling analyses for **(A)** healthy subjects, **(B)** healthy first-degree relatives, and **(C)** schizophrenic patients. Arrows indicating the causal coupling direction from one time series to another, e.g., RESP ← BBI, indicating the causal information transfer from BBI to RESP. Coupling strength ranges from blue (no coupling) to red (maximum coupling). BBI, beat-to-beat intervals; RESP, time intervals between consecutive breathing cycles.

**FIGURE 2:**
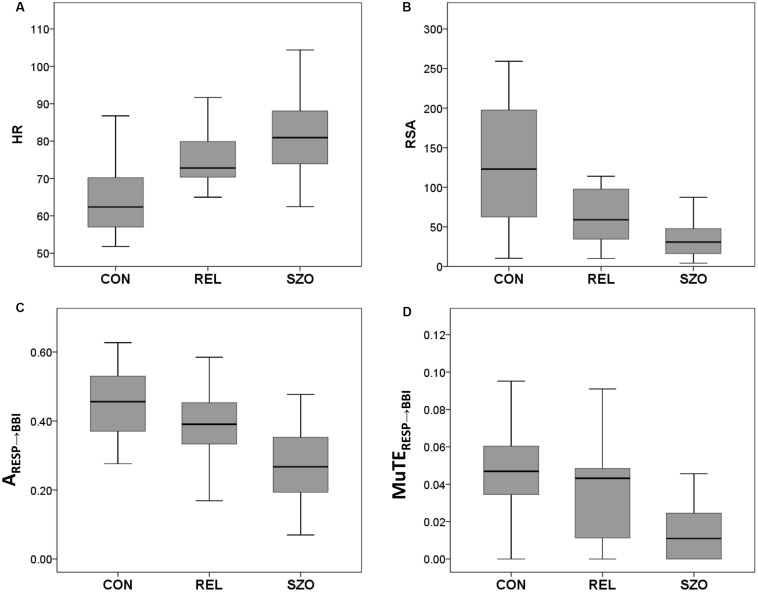
Box plots of significant cardiorespiratory indices from healthy subjects (CON), healthy first-degree relatives (REL), and schizophrenic patients (SZO) for **(A)** heart rate (HR), **(B)** respiratory sinus arrhythmia (RSA), **(C)** the coupling strength from normalized short-time partial directed coherence (NSTPDC) analysis from RESP to BBI, and **(D)** the coupling strength from multivariate transfer entropy (MuTE) analysis from RESP to BBI (BBI, beat-to-beat intervals; RESP, time intervals between consecutive breathing cycles). Boxes indicate data between 25th and 75th percentile with the horizontal bar reflecting the median.

NSTPDC analyses revealed a highly significant NF (CON: NF = −1.9 ± 0.2; SZO: NF = −1.0 ± 0.8) between SZO and CON. For CON, the NF was approximately −2, suggesting a strong unidirectional information transfer from RESP → BBI. For SZO the NF was approximately −1 pointing to a strong bidirectional information transfer with RESP as the driver (RESP → BBI). The coupling strengths were significantly different for both area indices (*A*_BBI → RESP_, *A*_RESP → BBI_) between both groups. In the case that BBI influenced RESP (*A*_BBI → RESP_), SZO demonstrated a higher coupling strength in comparison to CON. In the case that RESP influenced BBI (*A*_RESP → BBI_) we found a lower coupling strength for SZO compared to CON (Figures 1, 2).

MuTE showed similar results as NSTPDC, but with non-linear components, that in the case that BBI influenced RESP (MuTE_BBI → RESP_) higher coupling strength with non-linear components was present for SZO, and in the case that RESP influenced BBI (MuTE_RESP → BBI_) lower coupling strength with non-linear components was found for SZO in comparison to CON.

The uncoupling function quantifying the information transfer between HR and BF revealed an increased value for SZO in comparison to CON, pointing to stronger decoupling of the cardiac and respiratory system in SZO.

Highly significantly lower RSA values (RSA_P__2__V_) were found for SZO in compassion to CON.

All significant couplings were confirmed by surrogate analysis. No significant differences in linear and non-linear coupling indices were found between the groups for surrogate time series.

The results from phase randomization surrogate analysis revealed highly significant differences in all three NSTPDC indices whereas MuTE only showed significant MuTE_BBI → RESP_ (Table 3) comparing CON with SZO.

**TABLE 3:** Results of phase randomization surrogate analyses for cardiorespiratory couplings between patients suffering from paranoid schizophrenia (SZO), healthy first-degree relatives (REL), and healthy control subjects (CON).

	**Index**	**CON vs. SZO**	**CON vs. REL**	**SZO vs. REL**	**CON**			**REL**			**SZO**		
					**Median**	**[25–75]**		**Median**	**[25–75]**		**Median**	**[25–75]**	
Couplings	NF	***	n.s	***	−2.0	−2.0	−1.8	−1.8	−1.9	−1.5	−0.9	−1.5	−0.2
	*A*_BBI → RESP_	***	*	n.s	0.03	0.02	0.04	0.05	0.04	0.06	0.06	0.04	0.07
	*A*_RESP → BBI_	***	n.s	***	0.34	0.24	0.40	0.26	0.21	0.33	0.14	0.09	0.21
	MuTE_BBI → RESP_	**	n.s	n.s	0.002	0.000	0.008	0.005	0.002	0.012	0.008	0.003	0.016
	MuTE_RESP → BBI_	n.s	n.s	n.s	0.004	0.002	0.006	0.006	0.003	0.008	0.007	0.004	0.011

BB, beat-to-beat intervals; RESP, time intervals between consecutive breathing cycles; A, area from NSTPDC for identifying the coupling strength; MuTE, multivariate Transfer Entropy; p, univariate significance level: *<0.05, **<0.01, ***<0.004; n.s., not significant.

### Healthy First-Degree Relatives of Schizophrenic Patients vs. Healthy Subjects

Indices from cardiac variability demonstrated only a significant increased HR in REL compared to CON and consequently shortened mean value of the NN intervals (meanNN__*BBI*_).

Respiratory variability analyses did not demonstrate significant differences between REL and CON.

Results for cardiorespiratory couplings showed only significant different for RSA analyses with decreased RSA values (RSA_P__2__V_) for REL compared to CON (Table 2 and Figure 2).

All significant couplings were confirmed by surrogate analysis. No significant differences in linear and non-linear coupling indices were found between the groups for surrogate time series.

Phase randomization surrogate analysis showed significance for *A*_BBI → RESP_ comparing CON and REL (Table 3).

### Patients Suffering From Schizophrenia vs. Their Healthy First-Degree Relatives

Basic indices from HR and respiration did not contribute to a differentiation of these groups.

Results for cardiorespiratory couplings revealed highly significant differences for NSTPDC, CCE, and RSA analyses.

NSTPDC results demonstrated a highly significant NF value and *A*_RESP → BBI_ value between REL and SZO. Thereby, REL showed −1.7 indicating to a weak unidirectional coupling with RESP as the driver and BBI as the target variable. The coupling strength (*A*_RESP → *BBI)*_ was highly significant increased in REL compared to SZO (Figures 1, 2).

The uncoupling function revealed highly significant decreased value for REL in comparison to SZO, pointing to weaker decoupling (=stronger coupling) between the cardiac and respiratory system in comparison to SZO.

Significant higher RSA values (RSA_P__2__V_) for REL were found for REL compared to SZO (Table 2 and Figure 2).

All significant couplings were confirmed by surrogate analysis. No significant differences in linear and non-linear coupling indices were found between the groups for surrogate time series.

Phase randomization surrogate analysis for NSTPDC demonstrated a highly significant NF value and *A*_RESP → BBI_ value between REL and SZO (Table 3).

## Discussion and Conclusion

**FIGURE 3:**
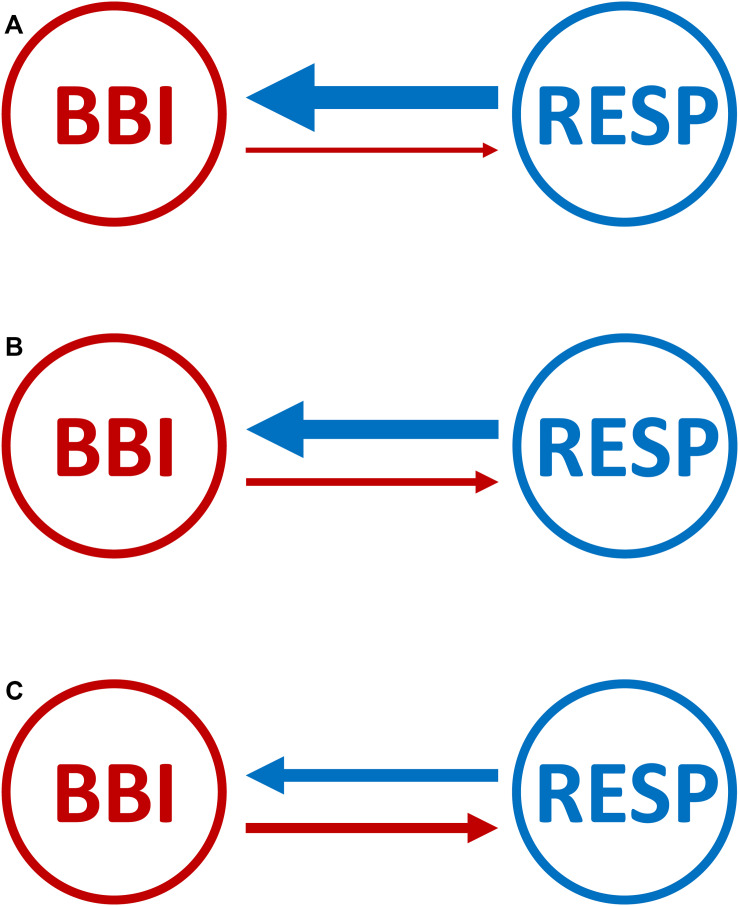
Graphical representation of the cardiorespiratory network structure with causal coupling strength and direction between the cardiac- and the respiratory system for **(A)** healthy subjects, **(B)** healthy first-degree relatives, and **(C)** schizophrenic patients. Arrows indicating the causal coupling direction from one time series to another. The thickness of the lines and arrows indicating the coupling strength and the direction between the two variables. The thicker the line the stronger the coupling strength (BBI, beat-to-beat intervals; RESP, time intervals between consecutive breathing cycles).

In our study, we found highly significant increased HR, reduced HRV, higher BF, and impaired cardiorespiratory couplings for schizophrenic patients compared to healthy control subjects. For SZO these couplings were characterized as bidirectional ones, with a driver-responder relationship from RESP → BBI, with weaker linear and non-linear coupling strengths when respiration influencing heart rate and with stronger linear and non-linear coupling strengths when HR influencing respiration. For the healthy first-degree relatives we found only significant increased HR and impaired RSA compared to healthy subjects (Figures 2, 3).

The variability analyses of basic heart rate indices are consistent with different studies that have shown an impaired sympathovagal tone in untreated schizophrenic patients (Mujica-Parodi et al., 2005; Boettger et al., 2006; Bär et al., 2007; Chang et al., 2010; Schulz et al., 2013c, 2015a). These results suggest an impairment of the ANS shown by a reduced HRV (sdNN__*BBI*_↓, meanNN__*BBI*_↓) expressed by a higher sympathovagal activation of the ANS. Furthermore, a predominant sympathetic activation for SZO (2.94 ± 2.28) was additionally confirmed by significantly increased LF/HF compared to CON (1.74 ± 1.57) and to REL (1.78 ± 1.15). Impaired cardiac regulation, which is one of the major contributors to changed sympathovagal balance, represented by higher basic HR in the first episode and during untreated conditions could clearly be demonstrated by several studies and seems to be a hallmark in schizophrenic patients. Bär et al. (2007) speculated that reduced HR seems to be highlighting that the cardiac system is not able to adapt to the different demands arising from posture or exertion, and moreover, that patients are at higher risk of developing arrhythmias. Valkonen-Korhonen et al. (2003) also demonstrated significantly reduced RMSSD and HF performance in psychotic patients and unchanged HRV in mental tasks than in healthy controls. They concluded that patients could not adjust HRV according to the task load. One could assume that acute psychosis state in these subjects leads to a restricted ability of the ANS to respond to external demands. Voss et al. (2011) also demonstrated reduced linear and non-linear HRV pointing to a higher sympathovagal activity in schizophrenic patients and their relatives. Moreover, the impairment of cardiac activity (complexity) confirms the assumption of an changed sympathovagal HR regulation in schizophrenia (Voss et al., 2009). Healthy first-degree relatives of patients showed only increased HR supported by other studies (Bär et al., 2010; Berger et al., 2010; Bär et al., 2012; Abhishekh et al., 2014).

Basic respiratory indices variability analysis showed significantly increased BF in SZO compared to CON supporting other findings dealing with untreated patients (Peupelmann et al., 2009; Bär et al., 2012; Schulz et al., 2012a). Here, it was shown that schizophrenia is accompanied by significantly shorter inspiration and expiration times and a higher BF. For healthy first-degree relatives we found no significant differences in respiratory activity compared to healthy subjects. This is in accordance to the findings of Bär et al. (2012), who only observed alterations in respiration for schizophrenia but not for relatives. They speculated that these findings could be a sign of excitement in critically diseased patients. In other study (Schulz et al., 2012a) we found significantly impaired respiratory variability and respiratory dynamics in schizophrenic patients, but neither for healthy first-degree relatives. This is a noteworthy fact that for schizophrenic patients and their first-degree healthy relatives comparable alterations in HRV (reduced) are present (Castro et al., 2009; Bär et al., 2010, 2012; Voss et al., 2010). These findings further leads to the assumption that an underlying disease-related genetic susceptibility of cardiac regulatory activity is obviously present in schizophrenia and relatives.

However, the entire cardiorespiratory system seems not to be affected, and that the dysfunction of the ANS appears to have a cardiac genetic basis. For example, it could also be shown that a genetic dependency of HRV was evident in healthy twins (Busjahn et al., 1998). In another study (Voss et al., 1996) HRV in the time- and non-linear dynamics domains found significant alterations between twin- and non-twin pairs (62, twin pairs: 30 monozygotic, and 32 dizygotic) leading to the assumption that there exists a genetic component in cardiac system in the generation of heart rate and its variability. Wang et al. (2009) investigated the heritability of HRV under stress and at rest and its dependency on ethnicity and gender (in 427 European and 308 African American twins). They found the same genes influenced HRV under stress and at rest independent of ethnicity and gender. Meda et al. (2014) investigated the genetic background of schizophrenia and its relatives studying the brain’s default mode network (DMN) of 296 schizophrenic patients (SZO), 179 unaffected first-degree relatives of SZO (SZREL) and 324 healthy subjects. They showed changes of functional connectivity in SZO and that these changes in DMN were selective only for SZREL familial, with genes regulating specific neurodevelopmental and transmission processes primarily mediating DMN discontinuity.

Personality anxiety has been shown to be associated with altered breathing alterations and BF (Masaoka and Homma, 1997, 1999). The authors found that a higher BF was not associated with metabolic factors and is coordinated with the limbic system and the respiratory drive (Masaoka and Homma, 2001). Boiten et al. (1994) found that alterations in breathing reflect the state a of emotional reaction connected with the requirements to react to emotional situations. Furthermore, symptoms of panic attacks and pulmonary patients overlap that panic anxiety highlights a cardiopulmonary disorder and that shortness of breath highlights an underlying anxiety disorder (Smoller et al., 1996). Respiratory changes may be explained by the fact that excitation disturbances in prefrontal are of the amygdala as assumed in paranoid schizophrenia could be responsible for the connection between psychopathology and alterations of respiration (Bär et al., 2012; Schulz et al., 2018, 2019). Therefore, chronic changes in HR and respiration in schizophrenia appear to be associated with cardiac dysfunction and not just a simple stress-related anxiety disorder (Schulz et al., 2015a).

Linear cardiorespiratory coupling analysis revealed a bidirectionally pronounced coupling direction (NF: −1.0) with respiration as the driver toward cardiac activity (RESP → BBI) in SZO vs. CON who demonstrating a more pronounced RSA regulation. The linear coupling from heart to respiration BBI → RESP (significantly increased in SZO) is supposed to be an RSA complementary biomarker as a reciprocal part of the cardiorespiratory interrelationship (Dick et al., 2014). Dick et al. (2014) stated that the joint interrelationship between the airways and the ANS as a function of gas exchange is emphasized by the fact that the ANS transmits information to the respiratory tract, which generates beat-to-beat changes, while the information transfer from respiration to the ANS is pronounced as a part of the RSA control loop.

Results from phase randomization surrogate analysis confirmed in general the underlying linear coupling structure for the directions BBI → RESP and RESP → BBI when considering NSTPDC results. But there was one exception, in the case when CON was compared with REL; we found in phase randomization surrogates a significant difference for *A*_BBI → RESP_ that was not present in the original time series. This can be a consequence of the low absolute values for the coupling strength.

For cardiorespiratory couplings with non-linear components significantly lower coupling strength was found in the direction RESP → BBI suggesting impaired non-linear regulatory pathways within the RSA-loop.

Phase randomization surrogates showed also significant differences between CON and SZO in the case of that BBI influenced RESP (MuTE_BBI → RESP_) as already found in the original time series. This means that the found difference in the coupling between CON and SZO (original time series) for the direction BBI → RESP was of linear nature without non-linear components. On the other side, we found no significant difference in coupling for the direction RESP → BBI in phase randomization surrogates (but in the original time series) pointing to a strong non-linear coupling behavior when RESP influenced BBI in the original time series.

In the study of Peupelmann et al. (2009), they found that the severity of schizophrenia is connected to alterations in breathing, and speculated that the vagal control within the brainstem does not work properly and leads to these findings. In the case that respiration transfers information toward the heart (RESP → BBI) is related to central respiratory driving mechanisms is respect to responses of the cardiac system (Faes et al., 2011). These impaired central respiratory driving mechanisms are assumed to be the cause of the impairments in the cardiac system in SZO (Schulz et al., 2015a, 2019). Bär et al. (2012) investigated cardiorespiratory couplings in control subjects compared to schizophrenic patients and their relatives. They observed impaired cardiorespiratory coupling, which was characterized by increased decoupling function (CCE) and complexity of cardiorespiratory couplings in schizophrenic patients. Williams et al. (2004) stated “that dissociation of amygdala prefrontal circuits and excitation leads to inhibition of signal processing of threat-related signals in SZO. In particular, dysregulation in the normal cycle of mutual feedback between amygdala work processes and autonomic regulatory activities is characterized by reduced amygdala activity and excessive excitation in these patients.” In addition, our results demonstrated that RSA is inhibited supported by other studies (Bär et al., 2012; Schulz et al., 2015a) which showed altered cardiorespiratory interactions and restricted RSA in untreated SZO. Thus, we speculate that impaired vagal control in the brain stem or restricted control pathways of higher centers is responsible for these results. In other study, we found that fractal structures of RSA were strengthened in SZO leading to the assumption that the rhythmic components of the RSA time series did fluctuated more randomly supporting the assumption that cardiac control in heart rate regulation agrees less with respiration in SZO leading to reduced RSA_P__2__V_ in SZO (Schulz et al., 2015a). We speculated that the impairment of cardiac regulation is not a stress-related excitation but more chronic and significant alterations in hear rate and breathing regulation (Schulz et al., 2015a). The significant alterations within the cardiorespiratory system seem clearly pointing to a disease-related hallmark in SZO and could reflect responses of the ANS during psychosis in acute schizophrenic patients. Due to, that relatives are not in the same “emotional and psychotic state” as patients it seems to be that the alterations within the cardiorespiratory system are closely connected to the emotions in SZO (Suess et al., 1980; Masaoka et al., 2001; Dimitriev et al., 2014; Jerath et al., 2015) and occur mainly only in this disease (Schulz et al., 2015b).

The novelty of this study, in contrast to our previous studies, is that in this study we were able to use a variety of methods from different domains, such as time domain, Granger causality and entropy domain. Especially, the application of two different causality approaches allowed us to assess coupling strength and the direction of the cardiorespiratory couplings. These features were not investigated and were possible so far. By determining causal relationships, it is now possible to understand how the cardiorespiratory system works in these patients. Schulz et al. (2015b), we applied and tested the introduced high-resolution joint symbolic dynamics approach to determine if with this approach a differentiation of the three groups is possible and to assess short-term non-causal couplings. We found a significantly altered heart rate pattern, respiratory pattern and cardiorespiratory couplings in SZO and only marginal alterations for REL group comparison to CON. Here, in this study, we are able to determine the causality of regulatory systems (heart and respiration) for these participants giving new insides of autonomic control.

For schizophrenia, this question has not yet been clarified as to what the defined working mechanisms are that are responsible for the obvious dysregulation of the ANS, since the number of brain areas (cortical, subcortical, and brain stem) is involved in autonomous regulatory processes. To sum up, we demonstrated a significantly impaired heart- and respiratory regulation expressed in their variability and dynamics and an impaired cardiorespiratory interactions in schizophrenic patients, and only a significantly altered heart rate regulation in healthy first-degree relatives. These results are consistent with previous studies, which also showed reduced HRV in schizophrenia and its first-degree healthy relatives, which clearly indicate underlying disease-related genetic vulnerability of the cardiovascular system (particularly within the cardiac subsystem). In schizophrenia, the results might be a result of lower vagal control within the brain stem, impaired communication between the brain stem and higher centers, or panic and anxiety-related alterations in the brain stem during the acute psychosis state in SZO (Schulz et al., 2018, 2019). Moreover, relatives do not seem to be in the same emotional and psychotic state as their sick schizophrenic relatives. As a result of the fact that in relatives only the cardiac system seems to be affected, it explains that the cardiorespiratory couplings are not significantly altered compared to the sick relatives (schizophrenia). Therefore, it seems that the alterations within the cardiorespiratory system and the linkages between the related subsystems that are apparent in schizophrenia are closely related to psychotic emotions that are evident during the acute phase of this disease (Schulz et al., 2015b), highlighted by alterations within the cardiac- and the respiratory systems. The interrelationship between the autonomous nerves system (cardiovascular and cardiorespiratory) in neuropathological diseases and the associated central control mechanisms are still not fully addressed in research.

## Data Availability Statement

The raw data supporting the conclusions of this article will be made available by the authors, without undue reservation, to any qualified researcher.

## Ethics Statement

The studies involving human participants were reviewed and approved by the local ethics committee of the Jena University Hospital. The patients/participants provided their written informed consent to participate in this study.

## Author Contributions

SS analyzed and interpreted the data, wrote the article, and final approval of the version to be published. K-JB conceived and designed the study, collected and assembled the data, interpreted the data, critically revised the article for significant intellectual content, and reread the final version prior to its publication. AV interpreted the data, did a critical revision of the article for its significant intellectual content, and reread the final version prior to its publication. JH did a critical revision of the article for its significant intellectual content, and reread the final version prior to its publication.

## Conflict of Interest

The authors declare that the research was conducted in the absence of any commercial or financial relationships that could be construed as a potential conflict of interest.
